# Popular Weather Signs

**Published:** 1874-04

**Authors:** 


### POPULAR WEATHER SIGNS.

Would it not serve a useful purpose if some scientific meteorologist were to gather into a mass the various weather signs—whether valuable or not—treasured by the farmers and other common sense people of the country, and then sift them, so that those of real value may have their proper influence, and those which are merely fanciful may cease to mislead?

That there are weather signs in abundance everybody knows; that the greater part of these signs are utterly valueless, every person of intelligence can testify; yet that they do practically influence the time and mode of the planting of crops, and of their after culture, will be acknowledged by many who would not be suspected of the folly, and who can give no other reason for it than the force of habit.

"We are going to have a dry month," said a farmer the other day.

"How do you know?" he was asked.

"By the Indian's sign of the new moon," he replied. "Its horns hung so sloping that they could hold no water."

His companion laughed. "Why, that's my Injun sign for a wet moon. The horns slope so that they let loose all the water."

The sign in the one case was no doubt as prophetic as in the other.

"Always plant your potatoes in the dark of the moon if you wish to have a full crop," I heard my neighbor say. "But never kill your pork, nor boil your soap at such a time, unless you are willing to have them shrink to nothing."

"What is your authority for this?"

"I have always so heard," he answered, with some hesitation, "and always so practiced. Potatoes, you know, being roots, naturally love darkness; and soap and bacon—I suppose they take their cue from the state of the moon. The fact is, I only know that this is the old time rule."

"We are to have a frost on the 19th of May," said a farmer to me on the 5th of April.

I was shocked, for he looked wise and lugubrious, and a frost at that time in our latitude would have cost millions of dollars. I asked, "How do you know?"

"Because we had a fog on the 19th of March."

He saw me smiling and added, "I have heard this rule ever since I was a boy and it has never failed yet."

"The surest rule I know for foretelling the weather throughout the year," said a planter, possessed of at least a semi-collegiate education, "is to note the twelve days between new Christmas and old Christmas (from December 25 to January 6). The months of the ensuing year are apt to be wet or dry, cool or warm, according to the days corresponding." He seriously declared that for many years he had "pitched his crop" and ordered his plantation work under the guidance of this rule and found that it served well. No doubt, for that amount of time in advance, it was quite as good as any other rule in ordinary use.—*F. R. G., in Hearth and Home.*

